# Psychosocial Impact of the Vehicle-Ramming Attack on the Magdeburg Christmas Market in Germany: Protocol for the MD-Care Longitudinal Cohort Study

**DOI:** 10.2196/100387

**Published:** 2026-11-03

**Authors:** Emily Elizabeth Jacumin, Florian Junne, Sophie Peter, Caroline Rometsch, Rosa Butzlaff, Kristina Geue

**Affiliations:** 1 University Clinic of Psychosomatic Medicine and Psychotherapy, Otto von Guericke University Magdeburg, Leipziger Str. 44, Magdeburg, Saxony-Anhalt, 39120, Germany, 49 3916715018; 2 Partner Site Halle-Jena-Magdeburg, Deutsches Zentrum für Psychische Gesundheit, Magdeburg, Saxony-Anhalt, Germany; 3 Center for Behavioral Brain Sciences, Magdeburg, Saxony-Anhalt, Germany; 4 Department of Psychosomatic Medicine and Psychotherapy, Universitätsklinikum Tübingen, Tübingen, Baden-Wurttemberg, Germany

**Keywords:** mass violence incident, vehicle-ramming attack, mental health, health care use, crisis

## Abstract

**Background:**

On December 20, 2024, a vehicle-ramming attack occurred at the Magdeburg Christmas market, resulting in 6 fatalities and more than 300 injuries. Mass violence incidents have the potential to cause long-lasting psychosocial consequences for directly and indirectly affected individuals. However, the outcomes can vary depending on relevant risk and protective factors. To improve the psychosocial aftercare of mass violence incidents in Germany, more research is essential.

**Objective:**

The MD-Care study strives to examine the psychosocial impact of the vehicle-ramming attack on the Magdeburg Christmas market by investigating the levels, longitudinal trajectories, and associated factors and predictors regarding (1) psychosocial, mental health, and somatic burdens; (2) individual resources and protective factors; and (3) mental health service use and support needs.

**Methods:**

The MD-Care study is a prospective longitudinal epidemiological study with 3 measurement time points. Data were collected pseudonymously via the online platform LimeSurvey. The baseline survey (t1; 3-month follow-up to the incident) was completed from March to April 2025. The 6-month follow-up (t2) was completed from June to July 2025. Finally, the 15-month follow-up survey (t3) was filled out by participants from March to April 2026. The surveys comprise standardized as well as self-developed ad hoc items addressing the objectives of the study.

**Results:**

The t1 baseline survey was completed by 336 participants, of whom 131 reported being affected individuals. The t2 follow-up survey was completed by 256 (76.2%) participants, 110 of whom reported being affected (110/131, 84% retention of affected individuals). The t3 follow-up survey was completed by 193 (57.4%) participants, 84 of whom reported being affected (84/131, 64.1% retention of affected individuals). The average age of participants for t1 was 39.10 (SD 13.30) years, and 79.5% (267/336) of the t1 sample were women. No funding was received for the quantitative data collection of the MD-Care study. Data analysis started in October 2025 and is ongoing. The first results are expected to be published by the fourth quarter of 2026 or the first quarter of 2027.

**Conclusions:**

The MD-Care study may contribute to the evidence base on the psychosocial impact of mass violence incidents, with a particular focus on vehicle-ramming attacks. The findings may inform the development of psychosocial emergency care, long-term support structures, and preparedness planning. Dissemination to relevant political and public health stakeholders will support the translation of the findings into practice and policy.

## Introduction

On Friday, December 20, 2024, a man intentionally drove a car into the crowd at the Christmas market in Magdeburg, Germany. The attack resulted in 6 fatalities and more than 300 injuries [1]. Mass attacks on humans represent a critical public health issue, as they entail not only immediate physical harm but also acute and enduring psychosocial consequences [2,3]. These attacks can markedly impact people’s mental health, disrupt social functioning, and place considerable strain on the health care and support systems of affected individuals, as well as the broader community [4,5].

Empirical evidence shows that individuals directly affected by mass violence incidents exhibit a higher prevalence of acute stress reactions, depression and anxiety disorders, and posttraumatic stress disorder (PTSD) [6-8], a pattern that has also been observed among survivors of natural and industrial disasters. Indirectly affected individuals also experience a higher prevalence of negative mental health impacts than the general population [4]. Identified risk factors for the development of PTSD include direct exposure to threat, bereavement, repeated media exposure, preexisting mental health conditions [6], and being a woman [9]. Additionally, several acute disaster-related experiences have been linked to adverse mental and physical health outcomes, including personal or familial injury, exposure to life-threatening situations, intense fear or panic, and separation from family members [9]. Protective factors, including social support; not being severely injured; and coping strategies such as using humor, positive reframing, and withdrawing, have been shown to mitigate the long-term psychosocial impact of traumatic events [8].

Mass violence incidents can also have a significant psychosocial impact on those involved in the immediate response, including first responders and health care professionals [5,10]. This can lead to the second victim phenomenon: second victims are characterized as health care professionals who are affected by the consequences of unanticipated medical events [11,12]. Symptoms can be long-lasting and can include distress and sadness [12]. Depression and PTSD are among the most frequently reported outcomes among medical responders following a disaster [13]. Additionally, psychological and psychosomatic symptoms such as troubling memories, anxiety, and sleeping difficulties are prevalent in health care providers [14]. Contributing risk factors include inadequate social support, poor communication in the workplace, insufficient training, and maladaptive coping strategies [13]. Social support (eg, by coworkers), job satisfaction, and prior disaster training have all been shown to act as protective factors for health care workers [15-17].

Disaster outcomes are shaped by the interplay of various risk and resilience factors. Individual responses to highly adverse events can differ considerably depending on factors related to the context in which the disaster occurs, the individual’s direct exposure during the event, and the experiences and circumstances following the disaster [18].

The European Network of Traumatic Stress guidelines on postdisaster psychosocial care recommend providing general support and access to social, physical, and psychosocial support, while reserving specific mental health interventions for individuals for whom these are indicated following a comprehensive assessment [19]. A study exploring the level of consensus of experts within Europe [20] recommended that psychosocial care in the aftermath of disaster should provide practical and emotional support, clear information, strengthen social networks and coping, and offer targeted psychosocial care to those who need it. Despite broad agreement on standards for early psychosocial care, substantial gaps between recommended practice and actual implementation persist across the European Union, highlighting the need for greater investment in the development and implementation of evidence-informed guidelines [20].

Given the unpredictable and chaotic nature of mass casualty incidents, more research to support proactive planning is essential to reach a broad range of impacted groups, including survivors, first responders, and those indirectly affected, who may require timely psychosocial support [21]. To our knowledge, few studies have examined the effects and impact of vehicle-ramming attacks in Germany. This represents an important research gap, particularly as such attacks appear to be becoming more frequent. Since the attack at the Magdeburg Christmas market in 2024, there have been 4 more vehicle-ramming attacks in Germany alone: in Munich (February 13, 2025), Mannheim (March 3, 2025), Leipzig (May 4, 2026), and Berlin (July 25, 2026) [22,23]. A better understanding of the characteristics and consequences of these attacks could help inform preventive measures and improve emergency response and postincident management strategies. The more evidence available on vehicle-ramming attacks, the better equipped authorities and emergency services will be to adapt existing plans and develop more effective approaches to preparedness and response.

The following protocol outlines the design, methodology, and implementation of the MD-Care study. This study aims to address the outlined gap by conducting an integrated, longitudinal analysis of psychosocial distress, individual resources, and the provision of psychosocial care following the vehicle-ramming attack in Magdeburg. By identifying unmet support needs and gaps in psychosocial care, this study hopes to contribute to the advancement of evidence-based psychosocial interventions and to provide practice-oriented insights for improving emergency mental health response in the context of mass trauma.

## Methods

This study protocol was written in accordance with the STROBE (Strengthening the Reporting of Observational Studies in Epidemiology) guideline [24] (Checklist 1). This protocol paper was written after data collection was already in progress.

### Study Design

This is a prospective longitudinal epidemiological study (MD-Care) with 3 measurement time points (Figure 1), examining the psychosocial impact of the attack on the Magdeburg Christmas market in Germany. The primary aim of the study is to examine the levels, longitudinal trajectories, and associated factors and predictors across the following three domains: (1) psychosocial, mental health, and somatic burdens; (2) individual resources and protective factors; and (3) mental health service use and support needs.

**Figure 1.**
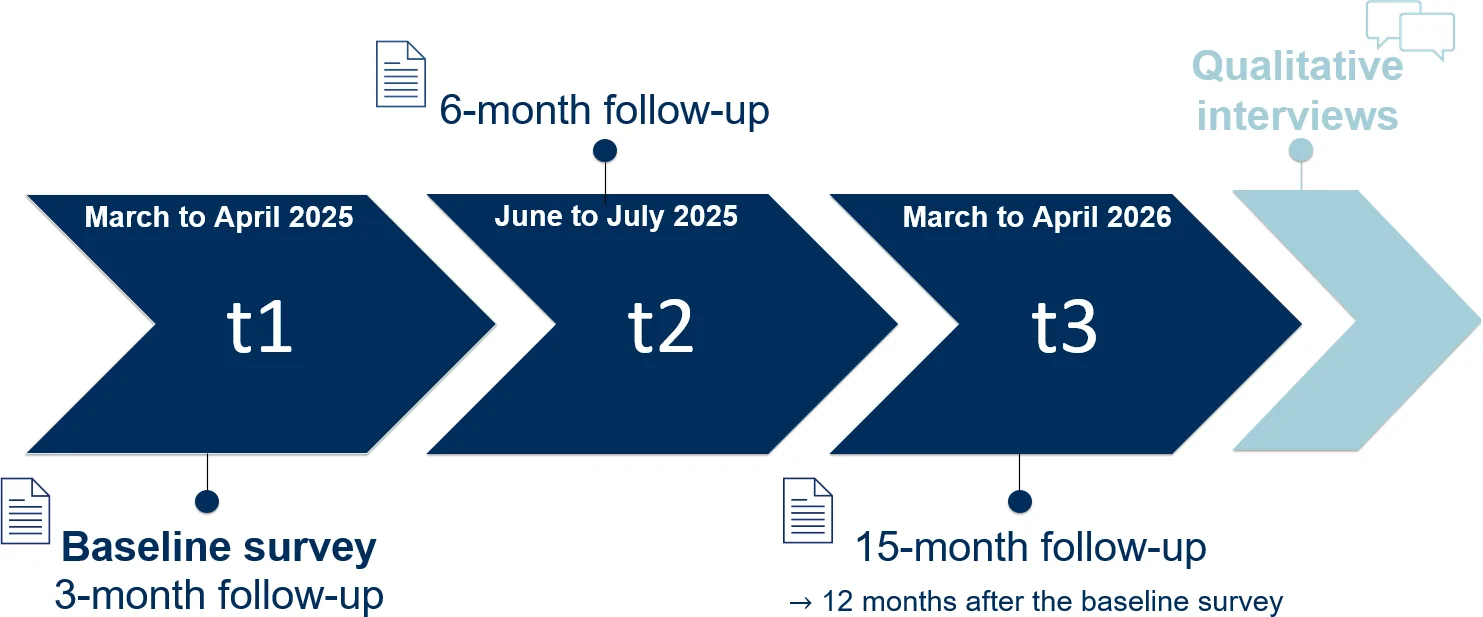
Measurement time points of the MD-Care study.

The overarching research aims and the 3 main domains of investigation were conceptualized prior to the start of data collection. However, more specific research questions and their methodological and analytical approach developed progressively over the course of the study. The findings are intended to highlight unmet care needs and guide the continued improvement of psychological emergency services.

### Procedure and Participants

To be eligible to take part in the study, participants had to be aged 18 years or older, have sufficient proficiency in the German language, and provide written informed consent. Affected individuals (eg, individuals directly affected by the attack, witnesses, first responders, relatives and acquaintances, and health care professionals) as well as unaffected individuals could take part in the study. Affected participants were asked to specify if they are directly affected as a survivor or on-site witness or indirectly affected as a relative or acquaintance of a directly affected person, a first responder (eg, paramedic, police officer, or firefighter), a member of the medical or psychosocial staff (eg, doctor or therapist), or other (asked to specify). Data were collected at 3 time points: 3 months after the attack, 6 months after the attack, and 15 months after the attack. From March to April 2025, individuals completed the baseline survey (t1). The second measurement point (t2) took place between June and July of 2025. The t3 survey was conducted between March and April 2026, 12 months after the baseline survey. Data were collected pseudonymously via the online platform LimeSurvey [25], with personal identifiers being managed by a data trustee. Recruitment channels for the baseline survey included the distribution of informational materials and study flyers (Multimedia Appendix 1) at the University Medical Center Magdeburg, Germany, as well as promotion of the study through social media and a TV feature to raise awareness of the study. No incentives were offered to participate in the study. For t2 and t3, participants were sent an email at an address which they had provided in t1, with an invitation and the link to participate in the next survey. The invitation emails were sent out a total of 3 times during the survey period as reminders to complete the survey. More details on the online surveys can be found in the CHERRIES (Checklist for Reporting Results of Internet E-Surveys; Checklist 2) [26]. Additionally, qualitative interviews will be conducted with a subset of participants after the completion of the t3 survey. At the end of the t2 survey, the participants were asked whether they would be willing to take part in an in-depth interview on the psychosocial effect of the attack. A total of 127 indicated their willingness to participate, 78 of whom were affected individuals. Recruitment for the qualitative interviews will start in the months following the t3 survey. Quantitative data collection was completed by May 2026.

### Monitoring of Participant Distress

As survey data were collected through a trusted third-party data management arrangement, individual responses could not be directly linked to participants by the study team for the purpose of actively monitoring distress during the surveys. However, at the beginning and end of each survey, all participants were informed that they could contact the study team at any time with questions or concerns arising from their participation. The interviews will be conducted by trained and professionally qualified interviewers who will be instructed to remain attentive to signs of distress during the interview. If an interviewer notices any signs of distress or concerns regarding a participant’s well-being, these will be addressed directly with the participant. Additionally, participants will be informed that they may withdraw from the interviews at any time, without giving a reason and without any consequences.

### Measures

The MD-Care surveys include items designed to capture the study’s primary aim of examining the levels, longitudinal trajectories, and associated factors and predictors across (1) psychosocial, mental health, and somatic burdens; (2) individual resources and protective factors; and (3) mental health service use and support needs. The questionnaires in the surveys addressing these themes were presented in a randomized order to minimize response bias. The surveys comprise self-developed (ad hoc) items as well as standardized questionnaires. Self-developed items include questions about the involvement in the attack, individuals’ subjective burden caused by the incident (eg, distress, psychological and physical well-being, media exposure, and experience of pain), as well as questions on the use, satisfaction, and evaluation of psychosocial care after the attack. The standardized questionnaires include instruments assessing depressive, somatic, and anxiety symptoms, along with individuals’ resilience, self-efficacy, social support, and sense of coherence. All instruments and items used are listed and described in Table 1. The table also specifies whether each instrument was applied within a cross-sectional or longitudinal design across the 3 measurement points.

**Table 1.** Overview of MD-Care assessment instruments used in the study at 3, 6, and 15 months after the attack.

Instrument	Reference	Construct assessed	Items and scale	t1	t2	t3
Psychosocial, mental health, and somatic burdens						
Subjective burden caused by the mass violence incidents	Ad hoc items	Measures individual’s distress and strain in different areas: personal strain, psychological and physical well-being, mental health history, experience of pain, and impact of media exposure	Various response formats (rating scales and open-ended responses)	✓	✓	✓
PHQ-9^a^; PHQ-2^b^ from t2 onward	Löwe et al [27]	Screens for depressive symptoms and assesses their severity over the past 2 weeks	9 items (2 items from t2 onward) to be rated on a 4-point-scale from “not at all” to “nearly every day”	✓	✓	✓
GAD-7^c^; GAD-2^d^ from t2 onward	Spitzer et al [28]	Measures the severity of generalized anxiety disorder symptoms and screens for probable cases of GAD	7-item (2-item from t2 onward) questionnaire to be rated on a 4-point-scale from “not at all” to “nearly every day”	✓	✓	✓
Perceived Stress Questionnaire	Levenstein et al [29] and Fliege et al [30]	Assesses current subjective stress across 4 domains: worries, tensions, enjoyment, and demands	20 items to be rated on a 4-point-scale from “almost never” to “usually”	✓		✓
Somatic Symptom Disorder–B Criteria Scale	Toussaint et al [31]	Evaluates cognitive, affective, and behavioral features associated with the somatic symptom disorder as defined in the *DSM-5^e^*	12-item self-report questionnaire on a 5-point-scale from “never” to “very often”	✓		
Resources and protective factors						
Questionnaire of Life Satisfaction	Henrich and Herschbach [32]	Assesses general quality of life and/or satisfaction	Consists of 9-item modules to be rated on a 5-point-scale from “dissatisfied” to “very satisfied” and “not at all” to “very”	✓	✓	✓
Generalized Self-Efficacy Scale	Jerusalem and Schwarzer [33]	Measures general optimistic self-beliefs, specifically an individual’s confidence in mastering difficult situations by attributing success to their own competence	10 items to be rated on a 4-point-scale from “not true” to “exactly right”	✓		
German social support questionnaire	Sommer and Fydrich [34] and Fydrich et al [35]	Measures social support as perceived or anticipated support	14 items (short form) to be rated on a five-point-scale from “does not apply at all” to “applies exactly”	✓		
Connor–Davidson Resilience Scale	Campbell-Sills and Stein [36] and Sarubin et al [37]	Measures the ability to cope with adversity	10 items to be rated on a 5-point scale from 0 (not true at all) to 4 (true nearly all of the time)	✓		✓
Sense of Coherence Scale	Singer and Brähler [38] and Schumacher et al [39]	Measures an individual’s sense of coherence, a global orientation that reflects how they view life as comprehensible, manageable, and meaningful	9 items to be rated on a 7-point scale with 2 anchoring verbal responses	✓	✓	
Psychological First Aid orientation pretest and posttest	World Health Organization [40]	Tests knowledge about the psychological reactions and coping strategies of people after crisis events	3 items, each answerable with yes or no			✓
Health care use and support needs						
Use of psychosocial care after the mass violence incident	Ad hoc items	Items regarding the use of professional psychosocial services since the attack	Various response formats (rating scales and open-ended responses)	✓		✓
Satisfaction and evaluation of psychosocial care after the mass violence incident	Ad hoc items	Assesses to what extent the treatment helped to alleviate the symptoms	Scale from 0 (“not at all”) to 10 (“very much”)	✓		
Support needs	Ad hoc items: adapted from the Short Form Supportive Care Needs Survey Questionnaire (Lehmann et al [41])	Subjective assessment of the extent to which participants required and/or had already received support in various areas (pain, fatigue, rumination, financial concerns, managing everyday activities, anxiety, depression, sadness, and sleep disturbances)	9 items to be rated on a 5-point-scale: 1 (no difficulties here; no need for support); 2 (already receiving support; no need for support); 3 (low; need for support); 4 (moderate; need for support); and 5 (high; need for support)	✓	✓	✓

a PHQ-9: Patient Health Questionnaire-9.

b PHQ-2: Patient Health Questionnaire-2.

c GAD-7: Generalized Anxiety Disorder-7.

d GAD-2: Generalized Anxiety Disorder-2.

e *DSM-5*: *Diagnostic and Statistical Manual of Mental Disorders *(*Fifth Edition*).

### Data Analysis

All analyses will be conducted using SPSS Statistics (IBM Corp) [42], the statistical software R (R Foundation for Statistical Computing) [43], and Microsoft Excel [44].

### Data Cleaning

Initially, the t1 dataset was processed and cleaned. This entailed the following steps. First, all participants who did not complete the entire survey from the first to the last page were deleted from the dataset. Second, 1 participant had to be excluded due to an unrealistically short survey completion time. Third, the response patterns were checked to examine whether participants selected the same response option repeatedly within the questionnaires. This was assessed by calculating LongString scores, that is, the maximum number of consecutive identical responses [45]. Cases with excessively long strings were flagged for further analysis. Upon closer examination and detailed plausibility checks, no participants were excluded in this step. These steps were completed with the t2 and t3 datasets.

### Statistical Analysis Plan

Descriptive statistics will be used to show participant characteristics and variables such as sociodemographic information and reported complaints and needs. This will include measures of central tendency (mean and median) and dispersion (SD and variance) to provide a detailed overview of the participants’ psychosocial status and well-being. For visualization purposes, data will be presented using boxplots and bar charts.

Inferential and multivariate statistical analyses will follow a primarily hypothesis-driven approach, testing already given theoretical mechanisms. Measures that are not based on any given theory and are new in this study will be analyzed in an exploratory manner to deduce new theories on psychological mechanisms when facing uncontrollable adversity, which should undergo confirmational analysis in future studies. Analyses will be organized into cross-sectional and longitudinal components to study structural associations and dynamical associations respectively.

Cross-sectional analyses will examine correlations and generalized linear models, considering the distributional properties of the outcome. Longitudinal analyses will use generalized linear mixed-effects models [46] and latent growth curve models [47] to assess contemporaneous effects and lagged-effects models [48] to assess effects across measurement time points; both analyses will take into consideration the multilevel data structure of this study. The latter allows for the exploration of the temporal ordering of effects across constructs, deriving possible causal mechanisms.

The analysis of missing data will depend on the model class [49-51]: full information maximum likelihood (FIML) will be used where applicable, whereas multiple imputation via chained equations will be used where FIML is not directly applicable. Longitudinal measurement invariance will be analyzed where applicable. Attrition patterns will be evaluated using the Little missing completely at random test [52] to assess whether data are missing completely at random, and by modeling dropout as a function of baseline demographics and affectedness, to evaluate whether the missing at random assumption underlying both approaches is plausible [53,54]. Table 2 shows more specifics of the statistical analysis plan.

**Table 2.** Specific statistical analysis plan.

	Planned analysis and methods
Levels	Descriptive analyses will include measures of central tendency (mean and median) and dispersion (SD and variance). Cross-sectional analyses will examine correlations and GLMs^a^, considering the given assumptions.
Longitudinal trajectories	Generalized linear mixed-effects models and latent growth curve models will be used to assess contemporaneous effects and trajectories over time, accounting for the multilevel data structure of the study.
Associated factors and predictors	Cross-sectional GLMs will be used to identify structural associations with levels within or across measurement occasions. Lagged-effects models will be used to assess effects across measurement time points (for measures with ≥3 measurement occasions), allowing exploration of temporal ordering of effects across constructs and derivation of possible causal mechanisms, while accounting for the multilevel data structure.

a GLM: generalized linear model.

Within each family of tests addressing a shared research question, *P* values (with a significance level of α=.05) will be adjusted for multiple comparisons. Sensitivity analyses will include a comparison of imputed versus complete-case estimates.

While longitudinal within-person analyses will constitute the primary approach to examining changes and temporal associations, between-group comparisons of affected and nonaffected individuals will be conducted for specific research questions where such comparisons are theoretically meaningful. For example, these analyses may examine whether the association between media exposure related to the event and psychological outcomes differs between affected and nonaffected individuals. Such comparisons will be considered complementary and interpreted cautiously, given the inherent differences between affected and nonaffected populations.

### Ethical Considerations

This study obtained approval from the Research Ethics Committee of the Medical Faculty at Otto von Guericke University Magdeburg (approval 02/25). All participants received an information notice and had the right to withdraw their consent at any time.

## Results

The baseline survey was completed by 336 participants (n=267, 79.5% women). Of those 336, a total of 131 (39%) were affected individuals. The mean age of the sample was 39.10 (SD 13.30; range 19-82) years. Table 3 shows the characteristics of the MD-Care participants for t1. Table 4 provides an overview of the different roles in which the affected individuals (n=131) were impacted. Participants could select more than one role, as an individual could be both directly affected by the attack and a relative or acquaintance of another individual directly affected by the attack.

**Table 3.** Sociodemographic characteristics of the MD-Care sample (n=336).

	Total (n=336)	Affected (n=131)	Not affected (n=205)
Age (y), mean (SD; range)	39.10 (13.30; 19-82)	—^a^	—
Age (y), n (%)			
18‐40	191 (56.9)	79 (60.3)	112 (54.6)
41‐60	120 (35.7)	47 (35.9)	73 (35.6)
>60	25 (7.4)	5 (3.8)	20 (9.8)
Gender, n (%)			
Women	267 (79.5)	106 (80.9)	161 (78.5)
Men	66 (19.6)	25 (19.1)	41 (20)
Nonbinary	3 (0.9)	0 (0)	3 (1.5)
Marital status, n (%)			
Single	116 (34.5)	47 (35.9)	69 (33.7)
In a relationship (not legally married)	90 (26.8)	36 (27.5)	54 (26.3)
Married or in a registered partnership	107 (31.8)	41 (31.3)	66 (32.2)
Other	23 (6.9)	7 (5.3)	16 (7.8)
Education, n (%)			
Secondary school certificate	48 (14.3)	20 (15.3)	28 (13.7)
High school diploma (university entrance qualification)	53 (15.8)	21 (16)	32 (15.6)
Vocational training	75 (22.3)	35 (26.7)	40 (19.5)
University degree	129 (38.4)	42 (32.1)	87 (42.4)
Other	31 (9.3)	13 (10)	18 (8.8)
Place of residence, n (%)			
Magdeburg	225 (67)	87 (66.4)	138 (67.3)
Near Magdeburg (within a 50 km range)	81 (24.1)	37 (28.2)	44 (21.5)
Elsewhere	30 (9)	7 (5.4)	23 (11.3)
Occupation, n (%)			
In training or studying	61 (18.2)	25 (19.1)	36 (17.6)
Employed part-time	63 (18.8)	21 (16)	42 (20.5)
Employed full-time	158 (47)	73 (55.7)	85 (41.5)
Other	54 (16.1)	12 (9.2)	42 (20.5)
Income^b^ (€^c^), n (%)			
<1000	42 (12.5)	16 (12.2)	26 (12.7)
1000-3000	153 (45.6)	62 (47.3)	91 (44.4)
3000-5000	87 (25.9)	30 (22.9)	57 (27.8)
>5000	38 (11.3)	17 (13)	21 (10.2)
On sick leave, n (%)	22 (6.5)	11 (8.4)	11 (5.4)

a Not applicable.

b Participants had the option of selecting “prefer not to say.”

c €1=US $1.12 as of October 9, 2026).

**Table 4.** Roles of affected individuals (n=131).

Roles of affected individuals^a^	Individuals, n (%)
Directly affected individuals	89 (67.9)
Relatives or acquaintances	20 (15.3)
First responders	29 (22.1)
Medical or psychosocial support staff	10 (7.6)

a Affected individuals were able to select more than one role (total answers=148).

The 6-month follow-up survey (t2) was completed by 256 (76.2 %) participants. Of those 256, a total of 110 were affected individuals. The 15-month follow-up survey (t3) was completed by 193 (57.4%) participants. Of those 193, a total of 84 were affected individuals (see Table 5). No funding was received for the quantitative data collection of the MD-Care study.

**Table 5.** Participant numbers at the 3 measurement time points.

	t1 (baseline survey; 3-month follow-up), n (%)	t2 (6-month-follow-up), n (%)	t3 (15-month-follow-up), n (%)
Total participants (n=336)	336 (100)	256 (76.2)	193 (57.4)
Affected individuals (n=131)	131 (100)	110 (84)	84 (64.1)

Data collection was completed in April 2026. Data analysis started in October 2025 (descriptive statistics for t1 sample characteristics) and is ongoing. Data on the use and evaluation of support services as well as reports on symptoms experienced after the attack have been analyzed and prepared for publication. All other analyses are yet to be conducted. The first results are expected to be published by the fourth quarter of 2026 or the first quarter of 2027.

## Discussion

The MD-Care study aims to examine how psychosocial and health burdens, protective factors, and mental health service needs and use vary over time and which factors are associated with these outcomes. Retention was strong across the 3 survey waves (256/336, 76.2% at t2 and 193/336, 57.4% at t3), with particularly high retention among affected participants (110/131, 84% and 84/131, 64.1%, respectively), despite the absence of financial compensation.

### Strengths and Limitations

Several strengths should be considered when evaluating the findings of the present study. First, the study included a relatively large participant number with satisfactory retention for t2 (256/336, 76.2%) and t3 (193/336, 57.4%). Second, the inclusion of 3 measurement time points allows for longitudinal within-person analyses. Third, to our knowledge, this is one of very few studies investigating the psychosocial impact of a vehicle-ramming attack in Germany, thereby addressing an important gap in the literature. Finally, the inclusion of ad hoc items with open-response formats provided participants with the opportunity to report experiences that may not be captured by standardized questionnaires, potentially allowing for the identification of previously overlooked aspects of the psychosocial impacts of an attack.

A key limitation of the MD-Care study is the nonprobability, convenience sampling strategy [55] based on open recruitment through flyers, social media, and television. This approach does not provide a defined sampling frame or response denominator, making it impossible to assess participation rates. Furthermore, participation was voluntary and based on self-administered surveys, which introduces motivation bias and may contribute to an overestimation of the prevalence of health problems. As a result, the findings should be interpreted with caution and cannot be generalized to the broader population of affected and unaffected individuals. In the immediate aftermath of a disaster, probability sampling is often not feasible. In Germany, strict data protection regulations generally prevent researchers from obtaining timely access to population registers or contact information for affected individuals.

As the attack on the Magdeburg Christmas market was an unexpected and unplanned event, the design of this study did not start until after the incident on December 20, 2024. The t1 baseline survey started in March 2025, allowing just over 2 months for the planning and implementation of the study. Consequently, certain aspects of the t1 survey could have benefited from more careful consideration. These limitations will be discussed, where relevant, in future publications on this study.

### Translation of Findings Into Public Health Practice and Policy

The findings of this study will be disseminated beyond the scientific community and made available to relevant political and public health stakeholders to support the further development of psychosocial emergency care and crisis response structures. In particular, the results will be communicated to political representatives and relevant regional and national stakeholders, including the Ministry of Science (Wissenschaftsministerium) of the German federal state of Saxony-Anhalt. Lessons learned from the response have already contributed to discussion regarding the optimization of mass casualty incident concepts and psychosocial emergency care in the region and may inform preparedness and response planning in other regions [56].

### Future Directions

For future work, it would be helpful to have precise and uniform definitions of who is considered affected vs not affected and directly affected vs indirectly affected, as, to our knowledge, no clear, established, and widely accepted definitions currently exist. A study examining the vehicle-ramming attack in Münster, Germany, on April 7, 2018 [57] considered individuals directly affected if they were survivors of the attack, witnessed it firsthand, or had a significant other who was a survivor or direct witness. Individuals were considered indirectly affected if they were not directly exposed but experienced distress after learning about the attack, such as concerns about their own or their family’s safety. The present study understands directly affected individuals as survivors or on-site witnesses of the attack and indirectly affected individuals as relatives or acquaintances of directly affected individuals, as well as first responders, medical or psychosocial staff, or others involved in the aftermath.

To improve psychosocial aftercare, it is vital to implement structured systems for identifying and monitoring affected individuals, as indicated in international guidelines [58-61]. Stene et al [58] concluded that standardized systems for registration, long-term follow-ups, and ongoing evaluation of the psychosocial care services can help ensure more consistent and effective responses to mass violence incidents. While this study does not focus on evaluating or implementing such standardized systems, the results may indirectly contribute ideas for future implementation. Malota et al [62] have already described a digital tool that helps distribute patients among hospitals more effectively. This reduced the risk of one hospital becoming too crowded. If emergency services (including psychosocial services) regularly use and practice with these types of systems, they may be better prepared for future large-scale emergencies.

### Conclusions

The MD-Care study may contribute to the evidence landscape of the psychosocial impact of mass violence incidents and specifically vehicle-ramming attacks in Germany. By systematically disseminating the resulting evidence to relevant stakeholders, the study aims to contribute to the evidence base for future public health policy, service planning, and emergency preparedness.

## Supplementary material

10.2196/100387 Multimedia Appendix 1 MD-Care study flyer.

10.2196/100387 Checklist 1 STROBE checklist.

10.2196/100387 Checklist 2 CHERRIES checklist.

## Abbreviations

CHERRIES: Checklist for Reporting Results of Internet E-Surveys
FIML: full information maximum likelihood
PTSD: posttraumatic stress disorder
STROBE: Strengthening the Reporting of Observational Studies in Epidemiology

## References

[1] Bell B (2026). Christmas market attacker jailed for life for murdering six in Germany. BBC.

[2] DiMaggio C, Galea S (2006). The behavioral consequences of terrorism: a meta-analysis. Acad Emerg Med.

[3] Salguero JM, Fernández-Berrocal P, Iruarrizaga I, Cano-Vindel A, Galea S (2011). Major depressive disorder following terrorist attacks: a systematic review of prevalence, course and correlates. BMC Psychiatry.

[4] Paz García-Vera M, Sanz J, Gutiérrez S (2016). A systematic review of the literature on posttraumatic stress disorder in victims of terrorist attacks. Psychol Rep.

[5] Wesemann U, Applewhite B, Himmerich H (2022). Investigating the impact of terrorist attacks on the mental health of emergency responders: systematic review. BJPsych Open.

[6] North CS, Pfefferbaum B (2013). Mental health response to community disasters: a systematic review. JAMA.

[7] Rigutto C, Sapara AO, Agyapong VI (2021). Anxiety, depression and posttraumatic stress disorder after terrorist attacks: a general review of the literature. Behav Sci (Basel).

[8] Newnham EA, Mergelsberg EL, Chen Y (2022). Long term mental health trajectories after disasters and pandemics: a multilingual systematic review of prevalence, risk and protective factors. Clin Psychol Rev.

[9] Norris FH, Friedman MJ, Watson PJ, Byrne CM, Diaz E, Kaniasty K (2002). 60,000 disaster victims speak: part I. An empirical review of the empirical literature, 1981-2001. Psychiatry.

[10] Rodriguez-Arrastia M, García-Martín M, Villegas-Aguilar E, Ropero-Padilla C, Martin-Ibañez L, Roman P (2022). Emotional and psychological implications for healthcare professionals in disasters or mass casualties: a systematic review. J Nurs Manag.

[11] Strametz R, Koch P, Vogelgesang A (2021). Prevalence of second victims, risk factors and support strategies among young German physicians in internal medicine (SeViD-I survey). J Occup Med Toxicol.

[12] Nydoo P, Pillay BJ, Naicker T, Moodley J (2020). The second victim phenomenon in health care: a literature review. Scand J Public Health.

[13] Naushad VA, Bierens JJ, Nishan KP (2019). A systematic review of the impact of disaster on the mental health of medical responders. Prehosp Disaster Med.

[14] Busch IM, Moretti F, Purgato M, Barbui C, Wu AW, Rimondini M (2020). Psychological and psychosomatic symptoms of second victims of adverse events: a systematic review and meta-analysis. J Patient Saf.

[15] Somville FJ, De Gucht V, Maes S (2016). The impact of occupational hazards and traumatic events among Belgian emergency physicians. Scand J Trauma Resusc Emerg Med.

[16] Schug C, Morawa E, Geiser F (2021). Social support and optimism as protective factors for mental health among 7765 healthcare workers in Germany during the COVID-19 pandemic: results of the VOICE study. Int J Environ Res Public Health.

[17] Perrin MA, DiGrande L, Wheeler K, Thorpe L, Farfel M, Brackbill R (2007). Differences in PTSD prevalence and associated risk factors among World Trade Center disaster rescue and recovery workers. Am J Psychiatry.

[18] Bonanno GA, Brewin CR, Kaniasty K, Greca AM (2010). Weighing the costs of disaster: consequences, risks, and resilience in individuals, families, and communities. Psychol Sci Public Interest.

[19] Bisson JI, Tavakoly B, Witteveen AB (2010). TENTS guidelines: development of post-disaster psychosocial care guidelines through a Delphi process. Br J Psychiatry.

[20] Te Brake H, Dückers M (2013). Early psychosocial interventions after disasters, terrorism and other shocking events: is there a gap between norms and practice in Europe?. Eur J Psychotraumatol.

[21] Stene LE, Fernandez A, Gindt M, Nachon O, Askenazy F (2023). Editorial: Recovering after terrorist attacks, large-scale accidents and other disasters: psychosocial care responses across countries. Front Psychol.

[22] Wollrab S, Knolle K (2026). Major vehicle-ramming incidents in Germany since 2016. Reuters.

[23] (2026). When cars become weapons – over 30 dead in Germany since 2018 [Article in German]. WELT.

[24] von Elm E, Altman DG, Egger M (2008). The Strengthening the Reporting of Observational Studies in Epidemiology (STROBE) statement: guidelines for reporting observational studies. J Clin Epidemiol.

[25] Klieve H, Beamish W, Bryer F, Rebollo R, Perrett H, Muyzenberg JV, Middleton H (2010). Knowledge in Technology Education: Proceedings of the 6th Biennial International Conference on Technology Education: Volume Two.

[26] Eysenbach G (2004). Improving the quality of web surveys: the Checklist for Reporting Results of Internet E-Surveys (CHERRIES). J Med Internet Res.

[27] Löwe B, Kroenke K, Herzog W, Gräfe K (2004). Measuring depression outcome with a brief self-report instrument: sensitivity to change of the Patient Health Questionnaire (PHQ-9). J Affect Disord.

[28] Spitzer RL, Kroenke K, Williams JB, Löwe B (2006). A brief measure for assessing generalized anxiety disorder: the GAD-7. Arch Intern Med.

[29] Levenstein S, Prantera C, Varvo V (1993). Development of the Perceived Stress Questionnaire: a new tool for psychosomatic research. J Psychosom Res.

[30] Fliege H, Rose M, Arck P, Levenstein S, Klapp BF (2009). PSQ - Perceived Stress Questionnaire [Article in German]. Leibniz-Institut für Psychologie (ZPID).

[31] Toussaint A, Murray AM, Voigt K (2016). Development and validation of the Somatic Symptom Disorder-B Criteria Scale (SSD-12). Psychosom Med.

[32] Henrich G, Herschbach P (2000). Questions on Life Satisfaction (FLZM): a short questionnaire for assessing subjective quality of life. Eur J Psychol Assess.

[33] Jerusalem M, Schwarzer R (2003). SWE. Skala zur allgemeinen Selbstwirksamkeitserwartung [Article in German]. Leibniz-Institut für Psychologie (ZPID).

[34] Sommer G, Fydrich T (1989). Soziale Unterstützung: Diagnostik, Konzepte, F-SOZU [Book in German].

[35] Fydrich T, Sommer G, Tydecks S, Brähler E (2009). Fragebogen zur sozialen Unterstützung (F-SozU): Normierung der Kurzform (K-14) [Article in German]. Zeitschrift Medizinische Psychologie.

[36] Campbell-Sills L, Stein MB (2007). Psychometric analysis and refinement of the Connor-Davidson Resilience Scale (CD-RISC): validation of a 10-item measure of resilience. J Trauma Stress.

[37] Sarubin N, Gutt D, Giegling I (2015). Erste analyse der psychometrischen Eigenschaften und Struktur der deutschsprachigen 10- und 25-Item Version der Connor-Davidson Resilience Scale (CD-RISC) [Article in German]. Zeitschrift Gesundheitspsychologie.

[38] Singer S, Brähler E (2007). Die “Sense of Coherence-Scale”: Testhandbuch Zur Deutschen Version [Book in German].

[39] Schumacher J, Wilz G, Gunzelmann T, Brähler E (2000). Die Sense of Coherence Scale von Antonovsky: teststatistische überprüfung in einer repräsentativen bevölkerungsstichprobe und konstruktion einer kurzskala [Article in German]. Psychother Psychosom Med Psychol.

[40] (2011). Psychological first aid: guide for field workers. World Health Organization.

[41] Lehmann C, Koch U, Mehnert A (2012). Psychometric properties of the German version of the Short-Form Supportive Care Needs Survey Questionnaire (SCNS-SF34-G). Support Care Cancer.

[42] Stehlik-Barry K, Babinec AJ (2017). Data Analysis with IBM SPSS Statistics: Implementing Data Modeling, Descriptive Statistics and ANOVA.

[43] Crawley MJ (2013). The R Book.

[44] Meyer DZ, Avery LM (2009). Excel as a qualitative data analysis tool. Field Methods.

[45] Meade AW, Craig SB (2012). Identifying careless responses in survey data. Psychol Methods.

[46] Bolker BM, Brooks ME, Clark CJ (2009). Generalized linear mixed models: a practical guide for ecology and evolution. Trends Ecol Evol.

[47] Duncan TE, Duncan SC (2009). The ABC’s of LGM: an introductory guide to latent variable growth curve modeling. Soc Personal Psychol Compass.

[48] Lucas RE (2023). Why the cross-lagged panel model is almost never the right choice. Adv Methods Pract Psychol Sci.

[49] Little R, Rubin D (2019). Statistical Analysis with Missing Data.

[50] Collins LM, Schafer JL, Kam CM (2001). A comparison of inclusive and restrictive strategies in modern missing data procedures. Psychol Methods.

[51] Graham JW (2009). Missing data analysis: making it work in the real world. Annu Rev Psychol.

[52] Little RJ (1988). A test of missing completely at random for multivariate data with missing values. J Am Stat Assoc.

[53] Twisk J, de Vente W (2002). Attrition in longitudinal studies. How to deal with missing data. J Clin Epidemiol.

[54] Diggle P, Kenward MG (1994). Informative drop-out in longitudinal data analysis. J R Stat Soc C Appl Stat.

[55] Stratton SJ (2021). Population research: convenience sampling strategies. Prehosp Disaster Med.

[56] Rometsch C, Geue K, Krüger J, Peter S, Junne F (2025). Erfahrungen der psychosozialen Notfallversorgung an der Universitätsmedizin Magdeburg nach dem Anschlag auf dem Magdeburger Weihnachtsmarkt [Article in German]. Notfall Rettungsmed.

[57] Morina N (2020). How am I doing compared to different standards? Comparative thinking and well-being following exposure to a vehicle-ramming attack. Eur J Psychotraumatol.

[58] Stene LE, Vuillermoz C, Overmeire RV (2022). Psychosocial care responses to terrorist attacks: a country case study of Norway, France and Belgium. BMC Health Serv Res.

[59] Bisson J, Tavakoly B (2008). The TENTS guidelines for psychosocial care following disasters and major incidents. European Society for Traumatic Stress Studies.

[60] Williams R (2008). Psychosocial care for people affected by disasters and major incidents: a model for designing, delivering and managing psychosocial services for people involved in major incidents, conflict, disasters and terrorism.

[61] Juen B, Warger R, Nindl S, Siller H, Lindenthal MJ, Huttner E (2015). The comprehensive guideline on mental health and psychosocial support (MHPSS) in disaster settings. Operationalising Psychosocial Support in Crisis.

[62] Malota M, Leitow R, Steinbrunner D, Kanz KG, Bogner-Flatz V, Hinzmann D (2026). Digital patient distribution and dynamic capacity management during a vehicle-ramming mass-casualty incident in Munich, Germany. Prehosp Emerg Care.

