# Supplementary material for: Psychosocial Impact of the Vehicle-Ramming Attack on the Magdeburg Christmas Market in Germany: Protocol for the MD-Care Longitudinal Cohort Study
Source: JMIR Res Protoc. 2026 Nov 3;15:e100387. doi: 10.2196/100387 (PMC13730500; doi:10.2196/100387)
Supplement: Supplementary file 1 [file resprot-v15-e100387-s001.pdf]

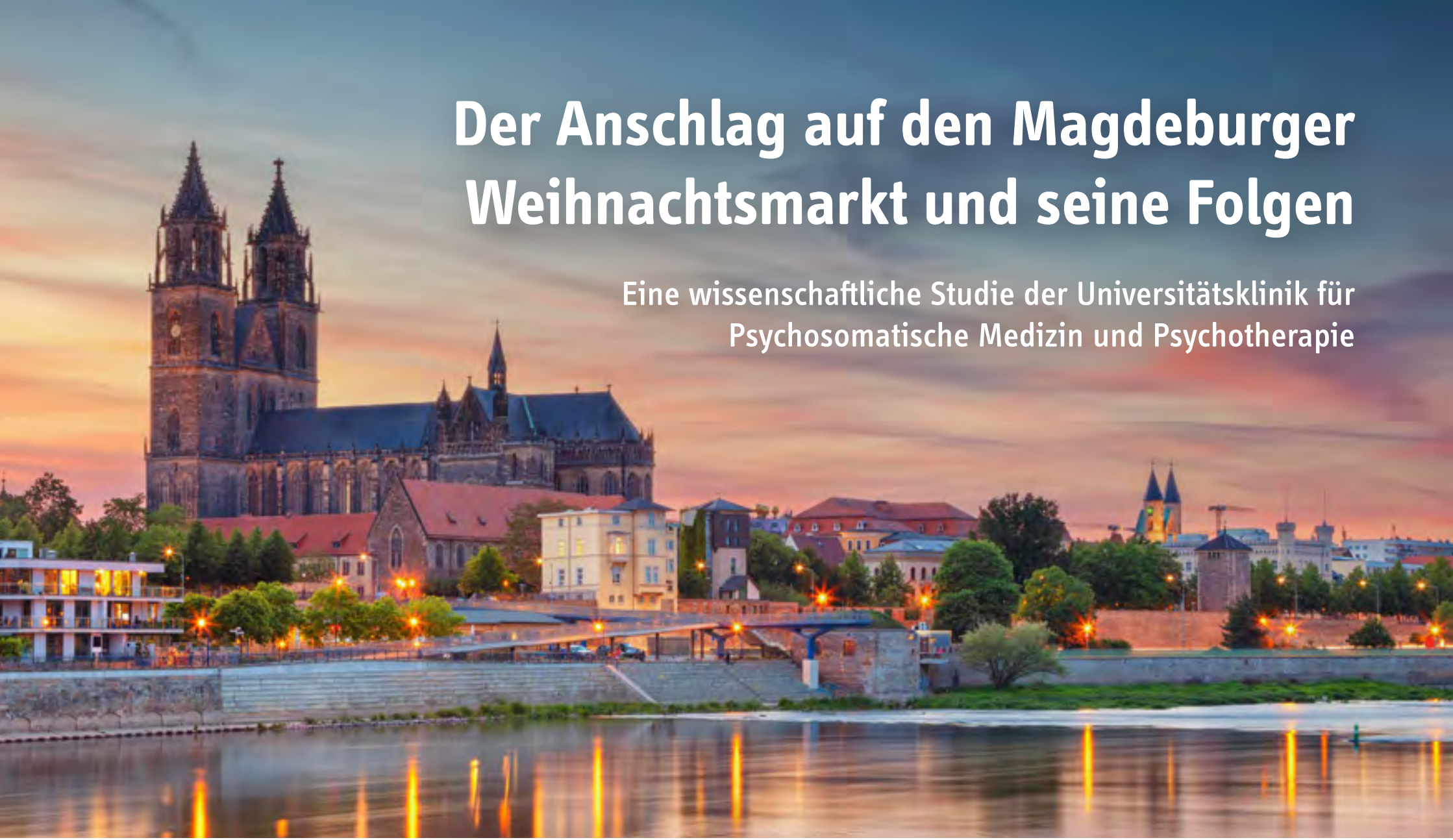

Der Anschlag auf den Magdeburger Weihnachtsmarkt und seine Folgen

Eine wissenschaftliche Studie der Universitätsklinik für Psychosomatische Medizin und Psychotherapie

UNIVERSITÄTSMEDIZIN
MAGDEBURG

Helfen Sie uns, Ihre Erfahrungen und Ihr Befinden nach dem Anschlag auf den Magdeburger Weihnachtsmarkt am 20.12.2024 besser zu verstehen. Nehmen Sie an unserer Online-Befragung teil und unterstützen Sie unsere Forschung.

Gerne können Sie diesen Flyer auch teilen!

Studienteilnahme unter:

https://limesurvey.ovgu.de/index.php/795818?lang=de

Oder scannen Sie diesen QR-Code:

Studienkontakt:

mdcare@ovgu.de

Um Ihre Anonymität zu wahren, wird die Befragung durch eine unabhängige Treuhandstelle durchgeführt.

UNIVERSITÄTSMEDIZIN

MAGDEBURG
