# Supplementary material for: Psychosocial Impact of the Vehicle-Ramming Attack on the Magdeburg Christmas Market in Germany: Protocol for the MD-Care Longitudinal Cohort Study
Source: JMIR Res Protoc. 2026 Nov 3;15:e100387. doi: 10.2196/100387 (PMC13730500; doi:10.2196/100387)
Supplement: Supplementary file 2 [file resprot-v15-e100387-s002.docx]

Appendix 1

STROBE (Strengthening the Reporting of Observational Studies in Epidemiology) Checklist

| **Item No.** | **STROBE recommendation** | **Manuscript location (page/section)** |
| --- | --- | --- |
| **Title and abstract** |  |  |
| 1a | Indicate the study’s design with a commonly used term in the title or the abstract. | Titel page – page 1 |
| 1b | Provide in the abstract an informative and balanced summary of what was done and what was found. | Abstract, pages 3-4 |
| **Introduction** |  |  |
| 2 | Explain the scientific background and rationale for the investigation being reported. | Pages 4-7 |
| 3 | State specific objectives, including any prespecified hypotheses. | Page 7, lines 23-26 and page 8, lines 1-2 |
| **Methods** |  |  |
| 4 | Present key elements of study design early in the paper. | Page 7, lines 16-23 |
| 5 | Describe the setting, locations, and relevant dates, including periods of recruitment, exposure, follow-up, and data collection. | Page 8, line 11 – page 9, line 15 |
| 6a | Give the eligibility criteria and the sources and methods of selection of participants. Describe methods of follow-up. | Pages 8, lines 12-13 & page 9, lines 4-9 |
| 6b | For matched studies, give matching criteria and number of exposed and unexposed participants. | Not applicable |
| 7 | Clearly define all outcomes, exposures, predictors, potential confounders, and effect modifiers. Give diagnostic criteria, if applicable. | Not applicable at this stage |
| 8 | For each variable of interest, give sources of data and details of methods of assessment (measurement). Describe comparability of assessment methods if there is more than one group. | Table 1 on pages 22-24 |
| 9 | Describe any efforts to address potential sources of bias. | Page 14, line 14 – page 15, line 4 |
| 10 | Explain how the study size was arrived at. | Page 11, lines 1-10 & page 13, lines 2-12 |
| 11 | Explain how quantitative variables were handled in the analyses. If applicable, describe which groupings were chosen and why. | Page 11, line 11 – page 12, line 26 |
| 12a | Describe all statistical methods, including those used to control for confounding. | Page 11, line 11 – page 12, line 26 |
| 12b | Describe any methods used to examine subgroups and interactions. | Page 12, lines 19-26 |
| 12c | Explain how missing data were addressed. | Page 12, lines 6-13 |
| 12d | If applicable, explain how loss to follow-up was addressed. | Page 12, line 3-4 |
| 12e | Describe any sensitivity analyses. | Page 12, line 17-18 |
| **Results** |  |  |
| 13a | Report numbers of individuals at each stage of the study (e.g., numbers potentially eligible, examined for eligibility, confirmed eligible, included in the study, completing follow-up, and analysed). | Page 13, lines 2-12 |
| 13b | Give reasons for non-participation at each stage. | Unknown due to online survey |
| 13c | Consider use of a flow diagram. | - |
| 14a | Give characteristics of study participants (e.g., demographic, clinical, social) and information on exposures and potential confounders. | Table 3 on pages 25-27 |
| 14b | Indicate number of participants with missing data for each variable of interest. | This will be reported for the respective constructs in the corresponding following publications |
| 14c | Summarise follow-up time (e.g., average and total amount). | Page 8, lines 20-24 & figure 1, page 28 |
| 15 | Report numbers of outcome events or summary measures over time. | Not applicable at this stage |
| 16a | Give unadjusted estimates and, if applicable, confounder-adjusted estimates and their precision (e.g., 95% confidence interval). Make clear which confounders were adjusted for and why they were included. | Not applicable at this stage |
| 16b | Report category boundaries when continuous variables were categorized. | Not applicable at this stage |
| 16c | If relevant, consider translating estimates of relative risk into absolute risk for a meaningful time period. | Not applicable at this stage |
| 17 | Report other analyses done (e.g., analyses of subgroups and interactions, and sensitivity analyses). | Not applicable at this stage |
| **Discussion** |  |  |
| 18 | Summarise key results with reference to study objectives. | Page 13, lines 2-19 |
| 19 | Discuss limitations of the study, taking into account sources of potential bias or imprecision. Discuss both direction and magnitude of any potential bias. | Page 14, line 14 – page 15, line 4 |
| 20 | Give a cautious overall interpretation of results considering objectives, limitations, multiplicity of analyses, results from similar studies, and other relevant evidence. | Page 15, line 16 – page 16, line 20 |
| 21 | Discuss the generalisability (external validity) of the study results. | Page 14, lines 14-24 |
| **Other information** |  |  |
| 22 | Give the source of funding and the role of the funders for the present study and, if applicable, for the original study on which the present article is based. | Not applicable, not funding received |

Source: Elm, Erik von; Altman, Douglas G.; Egger, Matthias; Pocock, Stuart J.; Gøtzsche, Peter C.; Vandenbroucke, Jan P. (2008): The Strengthening the Reporting of Observational Studies in Epidemiology (STROBE) statement: guidelines for reporting observational studies. In: Journal of clinical epidemiology 61 (4), S. 344–349. DOI: 10.1016/j.jclinepi.2007.11.008.
