# Supplementary material for: Psychosocial Impact of the Vehicle-Ramming Attack on the Magdeburg Christmas Market in Germany: Protocol for the MD-Care Longitudinal Cohort Study
Source: JMIR Res Protoc. 2026 Nov 3;15:e100387. doi: 10.2196/100387 (PMC13730500; doi:10.2196/100387)
Supplement: Supplementary file 3 [file resprot-v15-e100387-s003.docx]

Appendix 3

CHERRIES Checklist for Reporting Results of Internet E-Surveys

| **Item Category** | **Checklist Item** | **Description for the Present Study** |
| --- | --- | --- |
| **Design** | Describe survey design | Non-probability, convenience sampling strategy  Target population: Individuals residing in Magdeburg, the surrounding region, or elsewhere in Germany, including both those directly or indirectly affected by the Magdeburg Christmas market attack and those not affected. |
| **IRB approval and informed consent process** | IRB approval | Ethical approval was granted by the Ethics Committee of the Otto-von-Guericke University Magdeburg (Approval No. 02/25) |
|  | Informed consent | The survey landing page provided information on the length of time of the survey, the data management by an independent data trustee, the study purpose, as well as the right to withdraw at any time and confidentiality. Additionally, participants were provided information on the investigators of the study and how to contact them regarding any questions. |
|  | Data protection | Data was collected pseudonymously, with personal identifiers being managed by an independent data trustee. The anonymous data sets were sent via e-mail to the researchers. These were then stored on Confluence, a collaborative documentation platform. Access is permission-based, so users can only view pages they have been granted access to. |
| **Development and pre-testing** | Development and testing | The baseline survey was developed within a two month time frame, in order to have the baseline assessment as close to the Christmas market attack as possible. Survey items and questionnaires were added with the main objectives in mind. The finished survey was then completed by five team member who gave feedback on any errors they had found and on how long it took them to complete the survey. Adjustment were made to implement the feedback before distributing the link to possible participants. |
| **Recruitment process and sample** | Open survey versus closed survey | Open survey |
|  | Contact mode | Initial contact for the baseline survey was made through links on social media and QR-codes on flyers. For follow up surveys (t2 and t3) the participants from t1 were sent the survey links by e-mail by our data trustee. |
|  | Advertising the survey | Study flyer (see Appendix 2), social media (e.g. Instagram and sharing the link via WhatsApp), a TV feature to raise awareness of the study. |
| **Survey administration** | Web/E-mail | The survey was administered via the online platform LimeSurvey. |
|  | Context | The study flyers were distributed by employees of the Clinic of Psychosomatic Medicine and Psychotherapy of the University Hospital in Magdeburg. It was handed out to family, friends and acquaintances as well as patients within the clinic. The online link to the survey was similarly distributed through social media channels. This method may have pre-selected the sample to some extent. For example, the survey probably reached more individuals who sought support in the specialized psychosomatic outpatient clinic for affected individuals than any other support service. |
|  | Mandatory/voluntary | Voluntary |
|  | Incentives | No incentives were offered |
|  | Time/Date | t1: March to April 2025, t2: June to July 2025, t3: March to April 2026 |
|  | Randomization of items or questionnaires | Items were presented in a randomized order so that questionnaires assessing the same construct (e.g., protective factors or mental health symptoms) did not appear consecutively. However, each participant was presented the survey items in the same order. |
|  | Adaptive questioning | Adaptive questioning was employed throughout the surveys using conditional branching. Follow-up questions were displayed only when applicable based on participants’ responses to previous items, reducing both the number and complexity of questions presented to each participant. |
|  | Number of items | The number of items varied according to the questionnaire section and use of conditional branching. The number of items per page ranged from 2 to 20. |
|  | Number of screens (pages) | t1: 17 pages; t2: 10 pages, t3: 12 pages |
|  | Completeness check | Page-level completeness checks were used to ensure that mandatory items were completed before participants could proceed to the next page. For items where a response could reasonably be withheld or was not universally applicable, participants could select “Prefer not to say” or “Not applicable” as valid response options. |
|  | Review step | Participants were able to review and modify their responses by navigating back to previous pages using the survey navigation buttons. Previously entered responses were retained when moving between pages. A separate final review step displaying a summary of responses before submission was not implemented. |
| **Response rates** | Unique site visitor | Not applicable |
|  | View rate | Not applicable |
|  | Participation rate | Not applicable |
|  | Completion rate | Not applicable |
| **Preventing multiple entries** | Cookies used | Cookies were not used for assigning user identifiers or preventing multiple entries. |
|  | IP check | IP addresses were not collected or analyzed for duplicate detection, and no IP-based access restrictions were applied. |
|  | Log file analysis | No automated log file analysis was performed. Potential duplicates were manually checked by comparing email addresses and participants IDs. No duplicate IDs were found. Duplicate email addresses were interpreted cautiously because one address may be shared by multiple individuals. t2/t3 were invitation-based, so no new duplicate entries could be generated through open access. |
|  | Registration | Not applicable |
| **Analysis** | Handling of incomplete questionnaires | Only completed questionnaires were analyzed. |
|  | Questionnaires submitted with atypical timestamps | Response times were monitored to identify potentially implausibly short completion times. For each survey wave (t1, t2, and t3), colleagues completed the survey under conditions in which they attempted to finish as quickly as possible. These completion times were used to determined realistic lower cut-off thresholds for each survey. Questionnaires completed below the respective cut-off times were considered invalid and excluded from analysis. |
|  | Statistical correction | No weighting procedures or propensity score adjustments were applied to account for potential differences between the sample and the target population. |

Note: Adapted from Eysenbach, Gunther (2004): Improving the quality of Web surveys: the Checklist for Reporting Results of Internet E-Surveys (CHERRIES). In: Journal of medical Internet research 6 (3), e34. DOI: 10.2196/jmir.6.3.e34.
